# Single Nucleotide Polymorphism in Cell Adhesion Molecule L1 Affects Learning and Memory in a Mouse Model of Traumatic Brain Injury

**DOI:** 10.3390/ijms25053043

**Published:** 2024-03-06

**Authors:** Haoyu Jiang, Anna O. Giarratana, Thomas Theis, Vini Nagaraj, Xiaofeng Zhou, Smita Thakker-Varia, Melitta Schachner, Janet Alder

**Affiliations:** 1 Neuroscience and Cell Biology, Rutgers Robert Wood Johnson Medical School, Piscataway, NJ 08854, USA; hj356@scarletmail.rutgers.edu (H.J.); giarraan@scarletmail.rutgers.edu (A.O.G.); zhoux1@rwjms.rutgers.edu (X.Z.); varia@rwjms.rutgers.edu (S.T.-V.); 2 Cell Biology and Neuroscience, Rutgers School of Arts and Sciences, Piscataway, NJ 08854, USA; theis@dls.rutgers.edu (T.T.); vn149@cabm.rutgers.edu (V.N.); schachner@biology.rutgers.edu (M.S.)

**Keywords:** lateral fluid percussion, L1CAM, single nucleotide polymorphism, cognitive

## Abstract

The L1 cell adhesion molecule (L1) has demonstrated a range of beneficial effects in animal models of spinal cord injury, neurodegenerative disease, and ischemia; however, the role of L1 in TBI has not been fully examined. Mutations in the *L1* gene affecting the extracellular domain of this type 1 transmembrane glycoprotein have been identified in patients with L1 syndrome. These patients suffer from hydrocephalus, MASA (mental retardation, adducted thumbs, shuffling gait, aphasia) symptoms, and corpus callosum agenesis. Clinicians have observed that recovery post-traumatic brain injury (TBI) varies among the population. This variability may be explained by the genetic differences present in the general population. In this study, we utilized a novel mouse model of L1 syndrome with a mutation at aspartic acid position 201 in the extracellular domain of L1 (L1-201). We assessed the impact of this specific single nucleotide polymorphism (SNP) localized to the X-chromosome *L1* gene on recovery outcomes following TBI by comparing the L1-201 mouse mutants with their wild-type littermates. We demonstrate that male L1-201 mice exhibit significantly worse learning and memory outcomes in the Morris water maze after lateral fluid percussion (LFP) injury compared to male wild-type mice and a trend to worse motor function on the rotarod. However, no significant changes were observed in markers for inflammatory responses or apoptosis after TBI.

## 1. Introduction

L1 cell adhesion molecule (L1CAM, hereafter abbreviated L1) has been shown to reduce the severe consequences of neurological diseases, such as multiple sclerosis [1], Parkinson’s [2], and Alzheimer’s [3] disease, in mouse and zebrafish models. The study of these models has led to the view that the disease-adverse phenotypes could be reduced by treatment with neural stem cells overexpressing L1, administration of recombinant extracellular domain of L1, function triggering L1 antibodies, and L1-derived peptide interacting with itself, i.e., homophilically with L1 [4].

One area of particular interest is the role of L1 in central nervous system injuries [5]. And while other neuronal adhesion molecules have been investigated in injury [4,6,7,8,9,10,11], the role of L1 in TBI in vivo has not been extensively explored [12,13,14].

Mutated L1 in humans leads to the so-called L1 syndrome, a rare X-chromosome-linked disease, which affects male offspring of mutation-carrying mothers, with varying disease phenotypes, ranging from mild to life-threatening symptoms [15,16,17]. While mutation-carrying heterozygous mothers appear mostly unaffected by the mutation, the male mutant offspring display the typical features of the L1 syndrome, which are most consistently stenosis of the aqueduct of Sylvius, hydrocephalus, agenesis of the corpus callosum, agangliogenesis (Hirschsprung’s disease), and kidney dysfunction (Kakut disease), and neurological phenotypes are cognitive deficits, spastic paraplegia, and mental diseases, such as autism and schizophrenia [15,16,17]. Our previous studies have demonstrated that single nucleotide polymorphisms (SNPs) in *brain-derived neurotrophic factor (BDNF)* and *ApoE* result in increased risk for worse behavioral and cellular outcomes in brain trauma in mouse models [18,19]; however, the role of mutations in *L1* in injury remains to be elucidated.

To advance our understanding of L1′s functions, we previously generated a mutant mouse that carries a point mutation that is found in two families. This novel L1 syndrome mouse, with an SNP in the X-chromosome-localized *L1* gene at aspartic acid at amino acid 201 (202 in humans) and here termed L1-201, displays a cell-surface-exposed L1. Histological and behavioral analyses of male L1-201 mice showed features of the L1 syndrome [16]. Expression of mutated L1 at the cell surface was verified in cultures of live neurons by cell surface biotinylation and immunofluorescence. In a first attempt to ameliorate the deficits of L1-201 neurons with regard to neuritogenesis and neuronal survival, L1 mimetics were applied to cultures of cerebellar neurons and found to neutralize these deficits to the extent that they became normalized in comparison to wild-type cells. L1-201 Schwann cells defective in process formation were also found to be normalized [16] and the role of various signaling pathways of the L1 mimetics has been explored [20]. Since L1 is a major player in improving recovery after injuries of the central nervous system, our goal is to study if this mutation in the *L1* gene affects the recovery after traumatic brain injury (TBI).

TBI is a serious and potentially life-threatening clinical problem, which is growing at an increasing rate [21]. Many who survive the initial injury suffer from long-term chronic motor, cognitive, and psychological issues [22]. However, doctors have noticed for several decades that some patients recover better than others after a TBI [23]. If we can identify a susceptibility factor, then that would be an important advancement in revealing the mechanisms through which TBI can induce deleterious effects and also advance our focus on specific therapeutics targeted to those vulnerable individuals. This variability in consequences after TBI may be attributed to the genetic differences that exist in the general population [24,25].

In the present study, the L1-201 mutant mice were used to examine histological, locomotor, and cognitive functions following traumatic brain injury and compared to wild-type littermates. We demonstrate that L1-201 mutations cause worse cognitive outcomes after lateral fluid percussion (LFP) injury compared to wild-type mice and a trend to worse motor function, suggesting the important role of this SNP on recovery after TBI.

## 2. Results

### 2.1. Following TBI, Injured L1-201 Mice Had Impaired Learning and Memory Relative to Injured WT Mice

**Figure 1:**
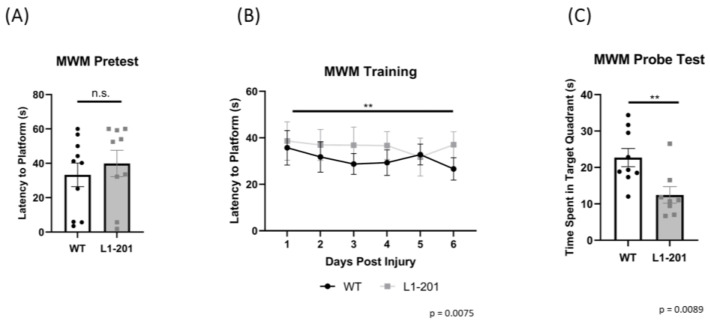
L1-201 injured mice have worse learning and memory relative to WT injured mice. (**A**) Pre-test phase showing average latency to locate platform ± SEM. (**B**) Average latency to find platform ± SEM in training phase from 14 to 19 DPI. (**C**) Average time spent swimming in the target quadrant in the probe test ± SEM at 20 DPI. n.s. = not significant, ** *p* < 0.01 L1-201 compared to WT. (**A**,**C**) Student’s two-tailed *t*-test; (**B**) two-way ANOVA, showing effect of genotype over time. n = 9, 10.

In the current study, we compared differences in outcomes between injured L1-201 and injured WT mice. We used the lateral fluid percussion (LFP) model of moderate injury since it shows face, construct, and predictive validities for TBI. First, we examined the effect of the L1-201 mutation on cognitive function since the LFP model has been shown to affect the hippocampal region [26] and L1 is expressed in the hippocampus [27,28]. Given that TBI is known to cause cognitive issues [29,30], we utilized the Morris water maze (MWM) test, which investigates spatial learning and memory. First, we ran the pre-test before the injury, in order to evaluate for any baseline differences in the assay between the two groups. In this study, we saw no significant difference between the two genotypes in the pre-test (Figure 1A). Next, we ran the training phase of the study starting 14 days post-injury (DPI) for 6 days. In this timeframe, we saw that the injured L1-201 mice had a longer latency to find the hidden platform relative to the injured WT mice over the course of the 6 days, showing the effect of genotype over time (*p* = 0.0075) (Figure 1B). These data suggest an impairment in spatial learning in the L1-201 mice after injury. Finally, we ran the probe test at 20 DPI, where we found that the injured L1-201 mice spent less time in the target NE quadrant than the injured WT mice (Figure 1C). These data suggest that the injured L1-201 mice have impaired spatial memory relative to injured WT mice. From these results, it is suggested that after injury, the L1-201 have both impaired spatial learning and memory relative to WT mice.

### 2.2. There Is a Trend for Injured L1-201 to Have Impaired Gross Vestibular Motor Function Relative to Injured WT Mice; However, There Is no Significant Difference Seen

**Figure 2:**
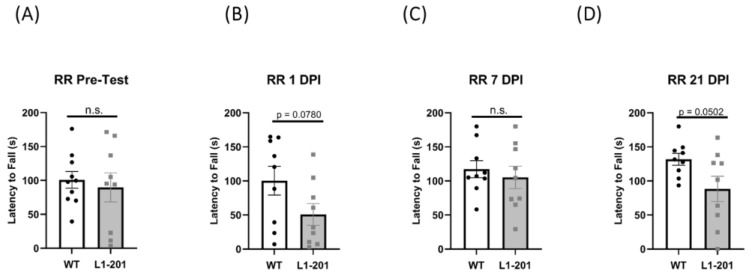
LFP injury induces no significant change in vestibular motor function in L1-201 mice relative to WT mice. (**A**) Quantification of latency to fall in the rotarod pre-test assay ± SEM (**B**) Quantification of the latency to fall in the rotarod assay ± SEM at 1 DPI, (**C**) 7 DPI, and (**D**) 21 DPI. n.s. = not significant, *p* values shown for (**B**,**D**), *p* < 0.05 is considered significant for L1-201 compared to WT. Student’s two-tailed *t*-test; n = 9, 10.

Given that, in addition to the hippocampus, our TBI LFP model also causes damage to the sensorimotor cortex [26], where L1 is expressed [31], we investigated the motor ability of these mice after injury. To assess gross motor ability, we used the rotarod test. We see no differences between genotypes in the pre-test (Figure 2A). We see a trend for the L1-201 mice to have impaired motor ability at 1 dpi (*p* = 0.0780), 7 dpi (*p* = 0.5656), and 21 dpi (*p* = 0.0502); however, we find no significant differences (Figure 2B–D).

### 2.3. There Are no Significant Differences in IBA1 between the Injured L1-201 and Injured WT Mice in either the Cortex or Hippocampus at 21 DPI

**Figure 3:**
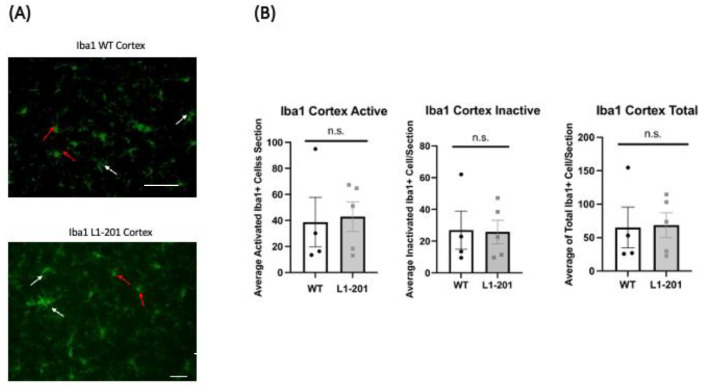
No change in ionized calcium binding adaptor molecule 1 (IBA1)-positive cells in the cortex of L1-201 injured mice compared to WT injured mice at 21 DPI. (**A**) Representative images of cortical sections at 21 DPI stained with IBA1. Resting microglia are indicated by white arrows and activated microglia are indicated by red arrows. Scale bars = 100 µm. (**B**) Average number of IBA1+ cells, broken down into activated and inactive categories by morphology as well as total, quantified per cortical section ± SEM. n.s. = not significant for L1-201 compared to WT, Student’s two-tailed *t*-test; n = 4, 5.

**Figure 4:**
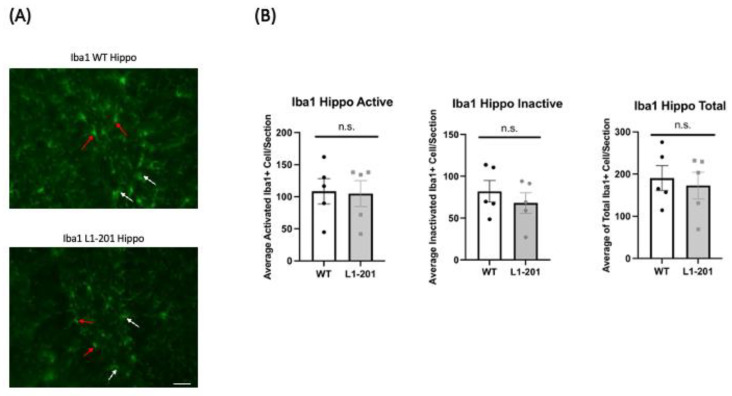
LFP injury causes no change in ionized calcium binding adaptor molecule 1 (IBA1)-positive cells in the hippocampus of L1-201 mice compared to WT mice at 21 DPI. (**A**) Representative images of hippocampal sections at 21 DPI stained with IBA1. White arrows indicate resting microglia, red arrows indicate activated microglia. Scale bars = 100 µm. (**B**) Quantification of the average number of IBA1+ cells, broken down into activated and inactive categories by morphology as well as total, per hippocampal section ± SEM. n.s. = not significant for L1-201 compared to WT, Student’s two-tailed *t*-test; n = 4, 5.

To elucidate cellular responses post-injury in mice, we conducted immunohistochemical staining at 21 DPI by perfusing the mice immediately following the final rotarod test. The study examined both focal and distal regions, specifically, both the ipsilateral cortex and the ipsilateral hippocampus, within our injury model. We investigated the neuroimmune response to repeated mild traumatic brain injury by analyzing microglia activation. Using IBA1 as a microglial marker, we differentiated activated microglia, characterized by bushy or ameboid shapes, from non-activated ones, identified by their ramified morphology [18,19]. At 21 DPI, we found that there was no significant difference in the cortex (Figure 3) and hippocampus (Figure 4) between the injured L1-201 and injured WT mice in active, inactive, or total IBA1+ cells.

### 2.4. There Are no Significant Differences in GFAP between the Injured L1-201 and Injured WT Mice in either the Cortex or Hippocampus at 21 DPI

**Figure 5:**
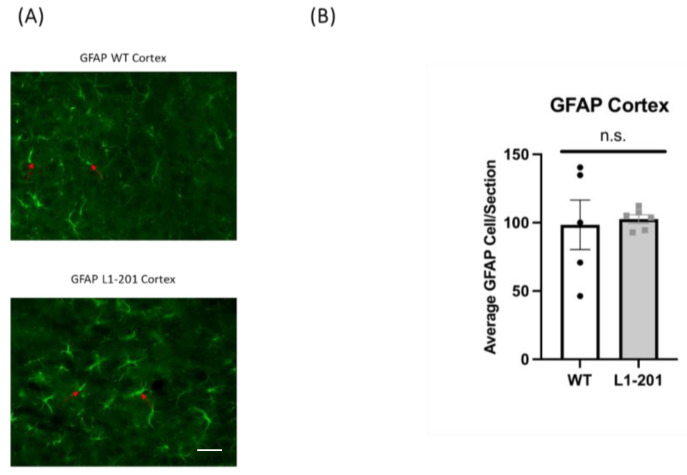
LFP injury causes no change in glial fibrillary acidic protein (GFAP)-positive cells in the cortex of L1-201 mice compared to WT mice at 21 DPI. (**A**) Representative images of cortical sections at 21 DPI stained with GFAP. Red arrows indicate representative positive cells. Scale bars = 100 µm. (**B**) Quantification of the average number of GFAP+ cells per cortical section ± SEM. n.s. = not significant for L1-201 compared to WT, Student’s two-tailed *t*-test; n = 4, 5.

**Figure 6:**
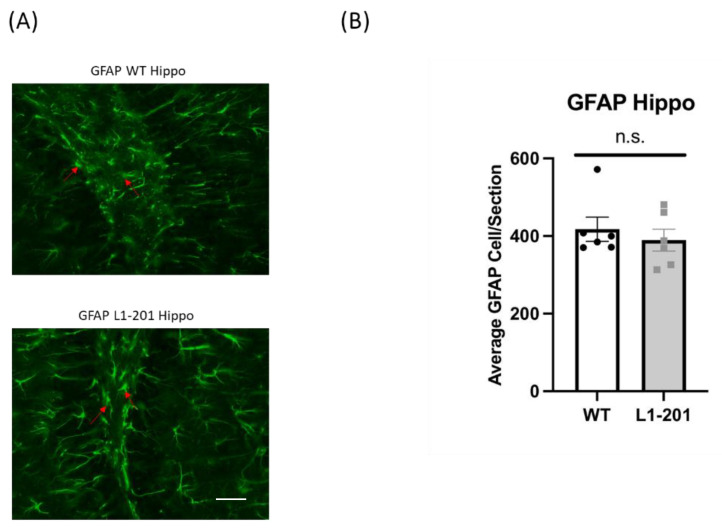
LFP injury causes no change in glial fibrillary acidic protein (GFAP)-positive cells in the hippocampus of L1-201 mice compared to WT mice at 21 DPI. (**A**) Representative images of hippocampal sections at 21 DPI stained with GFAP. Red arrows indicate representative positive cells. Scale bars = 100 µm. (**B**) Quantification of the average number of GFAP+ cells per hippocampal section ± SEM. n.s. = not significant for L1-201 compared to WT, Student’s two-tailed *t*-test; n = 4, 5.

Numerous studies have shown that glial proliferation occurs following injury, leading to glial scarring. This scarring hinders neuronal regeneration and impedes the injured brain’s recovery of its normal morphology and functionality [18,19,26]. In this study, we used glial fibrillary acidic protein (GFAP) as a marker for activated astrocytes to measure the extent of gliosis post-injury. At 21 DPI, we found no significant difference in the cortex (Figure 5) or the hippocampus (Figure 6) between the injured L1-201 and injured WT mice.

### 2.5. There Are no Significant Differences in Caspase between the Injured L1-201 and Injured WT Mice in either the Cortex or Hippocampus at 21 DPI

**Figure 7:**
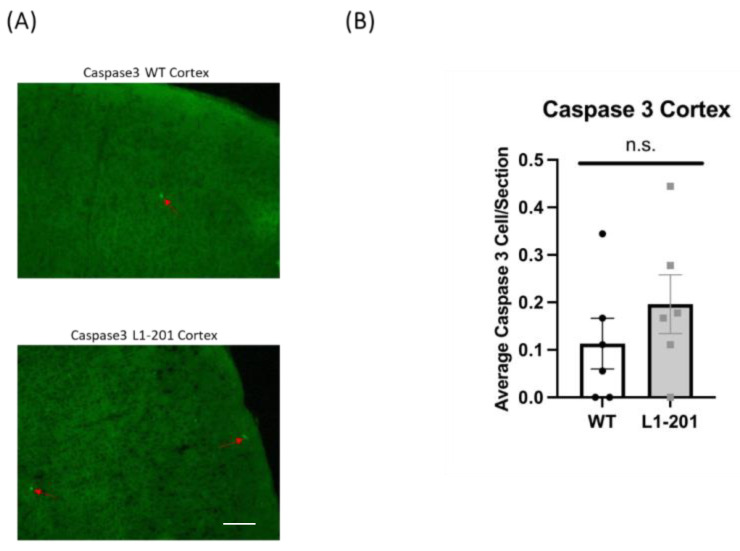
LFP injury causes no change in activated caspase-3-positive cells in the cortex of L1-201 mice compared to WT mice at 21 DPI. (**A**) Representative images of cortical sections at 21 DPI stained with caspase-3. Red arrows indicate representative positive cells. Scale bars = 100 µm. (**B**) Quantification of the average number of caspase-3+ cells per cortical section ± SEM. n.s. = not significant for L1-201 compared to WT, Student’s two-tailed *t*-test; n = 4, 5.

**Figure 8:**
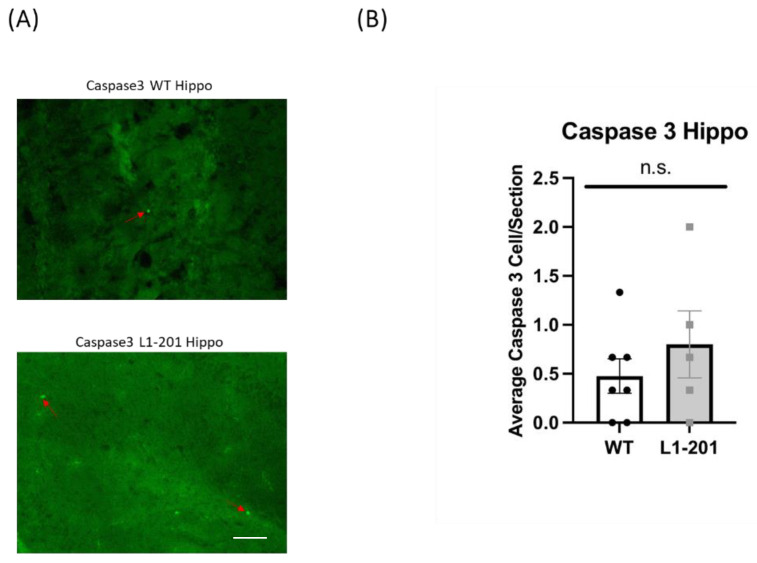
LFP injury causes no change in activated caspase-3-positive cells in the cortex of L1-201 mice compared to WT mice at 21 DPI. (**A**) Representative images of hippocampal sections at 21 DPI stained with caspase-3. Red arrows indicate representative positive cells. Scale bars = 100 µm. (**B**) Quantification of the average number of caspase-3+ cells per hippocampal section ± SEM. n.s. = not significant for L1-201 compared to WT, Student’s two-tailed *t*-test; n = 4, 5.

To assess apoptosis levels following injury, activated caspase-3 was employed as a marker, given the prevalence of neuronal cell death post-injury [18,19,26]. While the peak of activated caspase-3 is typically detected acutely after injury, there are a number of reports that caspase-3 levels are still elevated between 14 and 21 DPI [26,32,33,34,35,36]. At 21 DPI, we found no significant difference in the cortex (Figure 7) or the hippocampus (Figure 8) between the injured L1-201 and injured WT mice.

### 2.6. There Are no Significant Differences in the Number of Cells between the Injured L1-201 and Injured WT Mice in either the Cortex or Hippocampus at 21 DPI

**Figure 9:**
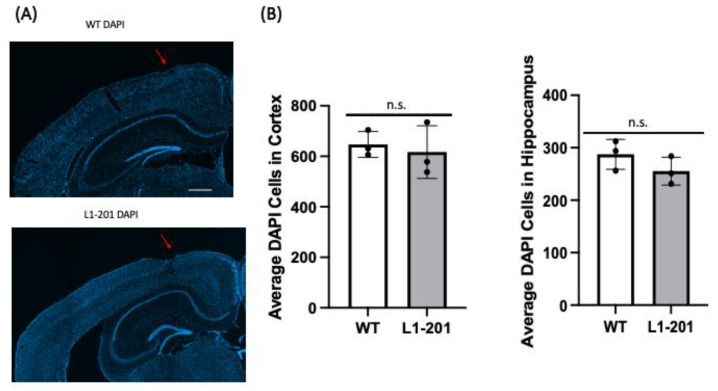
LFP injury causes no change in average number of cells in cortex or hippocampus of L1-201 mice compared to WT mice at 21 DPI. (**A**) Representative images of brain sections at 21 DPI stained with DAPI to label nuclei. Red arrows indicate site of craniectomy. Scale bar = 500 µm. (**B**) Quantification of the average number of DAPI-positive cells per cortical or hippocampal section ± SEM. n.s. = not significant for L1-201 compared to WT, Student’s two-tailed *t*-test; n = 3.

To assess for possible cell loss, total cell counts following injury were obtained using DAPI as a marker since it labels all nuclei. At 21 DPI, we found no significant difference in the cortex or the hippocampus (Figure 9) between the injured L1-201 and injured WT mice.

## 3. Discussion

Our study presents novel insights into the role of L1-201 on the recovery process post-TBI. We chose to focus on L1-201, given its documented beneficial effects in previous neural disease models [12,13,14]. The key finding from our research is the significant impairment in learning and memory outcomes in male L1-201 mice compared to their wild-type counterparts following LFP injury. This suggests a critical role of the L1-201 mutation in TBI cognitive recovery, emphasizing the importance of genetic factors in the variability in TBI outcomes. We did not detect any differences in baseline cognitive function between the L1-201 and the WT mice prior to injury, which suggests that the deficits we observed after injury are not a result of systemic issues in the mutant strain.

In addition, our results indicate no difference in baseline motor behavior in the L1-201 mice relative to WT mice prior to injury, suggesting that the mutation does not affect sensorimotor function. We did observe a strong trend towards worse motor function in L1-201 mice post-LFP, hinting that the mutation may exacerbate motor function after injury, although these findings were not statistically significant. This observation underscores the complexity of the relationship between genetic mutations like L1-201 and gross motor functional outcomes. In the past, previous studies have utilized more sensitive assays that investigate fine motor deficits, such as the balance beam assay [18,19]. Future studies may choose to utilize assays such as these to investigate the question of whether L1-201 mice have impaired motor function after injury relative to wild-type mice.

In our examination of the cellular markers after injury, we found that the neuroimmune response, probed by looking at microglia activation, showed no significant differences between the injured L1-201 and wild-type mice. This was also the case for markers of astrogliosis and apoptosis. We started with these markers because we previously published that an L1-function-blocking antibody increased the number of apoptotic cells in a cerebral ischemia–reperfusion rat model [12]. The absence of significant changes in inflammation, glial response, and neuronal cell death markers in the context of significant behavioral deficits in L1-201 mice is grounds for further investigation. One possibility is that the time point used for immunohistochemical studies is too long post-injury and that macrophage activation, astrogliosis, and apoptosis have already been attenuated 3 weeks after LFP. This is consistent with some our previous studies, where the largest difference in expression of these markers was detected at 1 DPI [18,19,26]. Even though the highest caspase-3 differences are observed at acute time points after injury, there are a number of studies, including our own findings, that demonstrate elevated caspase-3 levels are still present at 14 and 21 DPI [26,32,33,34,35,36]. To determine if cell loss had occurred prior to 21 DPI, total cell counts were performed and no difference in DAPI+ cells between WT and L1-201 injured mice was detected in either the cortex or hippocampus. These data support the conclusion that there are no differences between the two genotypes in cell survival prior to 21 DPI. Nevertheless, future studies should explore earlier time points post-injury for immunohistochemical differences between L1-201 and WT.

Alternatively, the lack of differences in markers of inflammation, astrogliosis, and apoptosis in the L1-201 mice relative to WT could indicate that the impact of the L1-201 mutation on cognitive and motor recovery post-TBI is mediated through alternative, less explored pathways, potentially involving synaptic integrity and neural network function. Previous studies have highlighted the role that changes in both inhibitory and excitatory synaptic function play after mild TBI [37]. In addition, neurotrophic signaling is well known to play a role in recovery after TBI, and future studies should investigate whether these factors are playing a role in the L1-201 mice [26,38,39]. The results from this paper highlight the need for further research focusing on synaptic and network-level changes post-TBI, especially in the context of genetic variations such as L1-201.

In summary, our study provides crucial evidence of the impact of the L1-201 genetic variation on recovery outcomes following TBI. Our data showcase the complexity of TBI pathology and the need for a deeper understanding of the genetic factors that influence recovery. Our findings also pave the way for future studies to explore the potential of targeting the L1 pathway, possibly through L1 mimetics that act as therapeutic agents [16,20], to improve recovery outcomes in TBI patients.

L1 plays an essential role in various regenerative-beneficial processes after nerve injury. Homophilic L1 interactions trigger cellular mechanisms such as neurogenesis, neurite outgrowth [5,40], synaptogenesis [27,41], myelination, and cell migration [42,43]. These homophilic interactions might be disrupted by the L1 mutation. Future studies should measure these regenerative parameters in L1-201 mice after TBI.

## 4. Materials and Methods

### 4.1. Animals

Adult male mice aged 10–12 weeks were used in all studies. Since male mice that carry this mutation are not fertile, heterozygous female mice were bred with wild-type male mice. Genotyping was performed as previously described [16] (briefly, ear punch biopsies were prepared for polymerase chain reaction (PCR) using the Phire Animal Tissue Direct PCR Kit (Thermo Fisher Scientific, Waltham, MA, USA). A 306 bp region in the *L1* gene was amplified by using L1_202 forward (FW) and reverse (Rev) primers (L1 202 FW: 5-TAG GAT CTA CTG GAT GAA CAG CA-3′; L1 202 Rev: 5′-AAA AC T TCT GGG ACT TAC TGG G-3′) and the following program: 98 °C for 5 min; 35 cycles of 98 °C for 10 s, 60 °C for 45 s, and 72 °C for 20 s; 72 °C for 7 min; 4 °C until further use. Subsequently, the amplified product was digested with the restriction enzyme DdeI (New England Biolabs, Ipswich, MA, USA) in CutSmart buffer (New England Biolabs) at 37 °C for 1 h. The amplification product of the wild-type mice was cleaved into one 150 bp and one 350 bp fragment. The mutated amplification product was not cleaved and remained as one 350 bp fragment. Hemizygous L1-201 males were used with their wild-type littermates as a control. Due to the L1-201 mutation being on the X chromosome, we only used male mice for this study. The housing for the mice was a 12 h light/dark cycle and water and food were available ad libitum. All procedures were approved by the Rutgers University Institutional Animal Care and Use Committee (IACUC) and were carried out in accordance with the NIH guidelines. To determine the appropriate sample size for experiments to reach 80% power, a power analysis was performed; the group sizes of n = 5–6 for histology, and n = 9–10 for behavioral tasks were selected to rigorously detect the magnitude differences expected between experimental groups based on our prior publications (α = 0.05) [18,19,26].

### 4.2. Lateral Fluid Percussion Injury

A fluid pulse resulting in rapid displacement of brain tissue is the basis for lateral fluid percussion injury. Here, we briefly summarize the detailed protocol previously published in [26]. Mice were anesthetized using 4–5% isoflurane in 100% O_2_ and then throughout the procedure were maintained on 2% isoflurane. A stereotaxic frame was used to secure the mice during the surgery. A 3 mm plastic disc was attached with Loctite glue (444 Tak Pak, Henkel Corporation, Rocky Hill, CT, USA) to the skull halfway between lambda and bregma, laterally on the right hemisphere. This disc acted a guide for the trephine (3 mm outer diameter) which is used to generate a craniectomy. Once the opening in the skull was created, a Luer-loc needle hub (3 mm inside diameter) was placed over the opening and secured using cyanoacrylate adhesive and dental acrylic (Henry Schein, Dublin, OH, USA). The animals were allowed to recover for 60 min and then re-anesthetized. The Luer-loc hub was then connected to the fluid percussion injury device (custom design and fabrication, Virginia Commonwealth University). The mice were monitored for sensitivity to tail pinch and immediately before full return of a normal breathing pattern, the pendulum was released to generate a ~1.2 ATM pulse (15 ms) of water on the brain dura. The righting reflex time was measured to determine moderate injury (4–10 min), after which the Luer-loc hub and dental acrylic were removed and the incision in the scalp was sealed with 3M Vetbond (Fisher Scientific, Waltham, MA, USA). The mice were returned to home cages and were individually housed to prevent damage to the injury site. Mice were monitored two times a day and if pain signals were observed, the veterinary staff were immediately contacted and analgesics applied. Humane endpoints were used for animals that showed signs of pain (e.g., decreased body condition, hunched posture, lethargy) or were not moving for hydration or food consumption. Pre-emptive analgesia injection of buprenorphrine (0.1 mg/kg SC) was given to avoid harm and suffering. During the surgery, the mice were anesthetized with isoflurane while they were in the stereotaxic apparatus. Prior to surgery commencing, anesthetic depth was determined by a lack of toe pinch response. Bupivicaine (0.025%) was applied as an anesthetic to the skull during the surgery procedure. Moreover, the eyes were protected with lubricant and the respiratory rate was monitored throughout the surgical procedure. Carprofen was delivered at 5 mg/kg, SC post-operatively, once per day if signs of pain or distress were observed (this was not necessary for any mice as part of this study).

### 4.3. Vestibular Rotarod Test

To examine the sensorimotor capabilities of the mice after LFP, the vestibular rotarod test was performed. The rotarod test utilized a 36 mm outer diameter rotating rod whose velocity was increased from 4 to 40 rpm over a maximum 180 s interval. Latency to fall off the rotating rod was used as a measure of motor function and balance. Each group consisted of nine to ten mice per genotype and treatment condition. To measure any baseline genotypic differences in motor function and to acclimate the mice to the behavioral paradigm, the mice were trained on the rotarod one day before the injury, using three trials separated by a one-hour inter-trial period. At 1, 7, and 21 days after lateral fluid percussion injury, each mouse underwent 3 trials separated by a one-hour inter-trial rest period. The same cohort of mice was used for each trial day. The person analyzing the data was blinded to the experimental group. The average latency to fall between cohorts was compared.

### 4.4. Morris Water Maze Test

To study spatial memory, the Morris water maze test was performed. Baseline differences in genotypic performance and acclimation to the paradigm were determined using a visible platform 1 day prior to the lateral fluid percussion injury. A circular pool (1 m diameter) with water made opaque using non-toxic white paint was used. For the pre-test, an escape platform made of clear plexiglass was made visible with a black and white rod. For the training period, mice were scored for their ability to use special cues to locate the platform that was now hidden in the northwest quadrant. The spatial cues consisted of black and white images on the room walls as well as geometric shaped cues positioned around the perimeter of the pool. Learning tests were started 14 days after the LFP injury and consisted of 4 trials per day for 6 days in a row. For each trial of testing, the mice were placed in the pool in randomized quadrants, and the time for the mouse to locate the platform was recorded. When the mouse found the platform the trial ended, and if the mouse did not find the platform after 60 s (max trial time) the mouse was placed on the platform. When the trial concluded, the mouse was left on the hidden platform for 15 s in order to facilitate learning consolidation. The mouse was then taken out of the pool and warmed on a heating pad for 10 min. On the 7th day (day 20 after LFP), memory was assayed using a probe test. The probe test consisted of the hidden platform being removed and then recording the time the mouse spent looking for the platform in the northwest quadrant. Nine to ten mice per group and condition were used. The individuals analyzing the data were blind to the group condition. A video-tracking system (EthoVision XT; Noldus Information Technology, Leesburg, VA, USA) was used for recording and collecting the data.

### 4.5. Immunohistochemistry

To assess markers of inflammation, astrogliosis, and apoptosis, immunohistochemistry was performed. Following the last behavioral test, at 21 days post-LFP, mice were perfused with 0.9% saline, followed by 4% paraformaldehyde. The brains were then incubated in 30% sucrose for at least 3 days for cryoprotection. Twenty µm thick slices throughout the anterior–posterior axis of the hippocampus and inclusive of the injured cortical area were sectioned using a Cryostat (Leica, Deerfield, IL, USA) in a 1:10 series. Microglial activation was assayed using an IBA1 antibody (1:2000, ab178846 Abcam, Boston, MA, USA) overnight followed by secondary Alexa Flour goat anti-rabbit 488 (1:200, Thermofisher Scientific, Waltham, MA, USA). Astrogliosis was visualized using glial fibrillary acidic protein (GFAP) antibody (1:1000, G9269, Sigma Aldrich, St. Louis, MO, USA) overnight followed by Alexa Fluor goat anti-rabbit 488 (1:200, Thermofisher Scientific, Waltham, MA, USA). Apoptotic cell death was determined by pretreating sections with 0.01 M citrate buffer at 90 °C followed by anti-cleaved caspase-3 antibody (1:1000, 9661, Cell Signaling, Danvers, ME, USA) overnight and then Alexa Fluor 594 goat anti-rabbit (1:200, Thermofisher Scientific, Waltham, MA, USA). To visualize nuclei, all slides were incubated in 4′,6-diamidino-2-phenylindole (DAPI) (1:1000 DAPI in PBS, Sigma, St Louis, MO, USA) followed by coverslipping with Fluoromount-G (Southern Biotech, Birmingham, AL, USA). A Leica microscope (Model DMIRB, Leica Microsystems, Buffalo Grove, IL, USA) was used to visualize the staining and for quantitation.

### 4.6. Quantification of Immunohistochemistry

Five to six mice per group (treatment and time point) were analyzed. The average number of positive cells per section was determined on the hemisphere ipsilateral to the injury. All of the sections for each brain were counted at 40× and analyzed for each biological replicate. For the cortical areas, starting at the dorsal midline and moving laterally, a total of 6 fields of vision were counted (3 along the dorsal aspect of the brain and 3 just ventral to that row). The dentate gyrus and CA1-CA3 regions were used for quantification of positive cells in the hippocampus. For quantification of DAPI staining 3 WT and 3 L1-201 brains were counted, with 4 sections per brain. For each section, 3 regions in the cortex and 3 regions in the CA1-3 area of the hippocampus were imaged at 20×. Cell counts per region were quantified using the Image J software (https://imagej.net/ij/, accessed on 29 December 2023). The individual performing the quantitation was blinded to experimental genotype and group.

### 4.7. Statistical Analysis

All data analysis was run through the GraphPad Prism Software (version 9.5.1). Student’s two-tailed *t*-test or one-way ANOVA followed by Fisher’s PLSD post hoc analysis was used for group comparisons. To be considered statistically significant, the *p* < 0.05 criterion must be met.

### 4.8. Animal Study

All procedures described were performed in accordance with the NIH guidelines and were approved by the Rutgers University Institutional Animal Care and Use Committee (IACUC). Protocol number TR202300115; approval date: 25 August 2023.

## Data Availability

Data is contained within the article.
